# Complications of Extended Pelvic Lymph Node Dissection in Patients Undergoing Minimally Invasive Radical Prostatectomy: Analysis and Risk Factors

**DOI:** 10.1155/2022/7631903

**Published:** 2022-10-22

**Authors:** Pedro Castro, Paulo B. O. Arantes, Yves M. R. Martins, Matheus N. M. Reis, Ana Paula Drummond-Lage, Alberto J. A. Wainstein

**Affiliations:** ^1^Faculdade de Ciências Médicas de Minas Gerais, 275 Ezequiel Dias Street, Belo Horizonte 30130-110, Brazil; ^2^Instituto Oncomed de Saude e Longevidade, Jose do Patrocinio Pontes Avenue, Belo Horizonte 30210-090, Brazil

## Abstract

**Background:**

The knowledge of risk factors and complications related to extended pelvic lymph node dissection (ePLND) during radical prostatectomy can help selecting patients who will benefit the most with lymph node dissection concomitant to radical prostatectomy.

**Materials and Methods:**

Retrospective cohort evaluating 135 patients with PC, with a high risk for lymph node metastasis, submitted to ePLND by a single surgeon between 2013 and 2019, performed either by the laparoscopic or laparoscopic robot-assisted approach. Data related to complications were properly recorded using the Martin's criteria and were classified by the Satava and Clavien–Dindo–Strasberg methods. Logistic regression was used to determine predictors of complications related to ePLND.

**Results:**

The mean number of lymph nodes removed was 10.2 ± 4.9, and in 28.2%, they were positive for metastasis. There were five intraoperative complications (4%), all in patients operated by laparoscopic approach. There were nine severe postoperative complications (7.3%), four of which occurred after postoperative day 30. Three patients (2.4%) had thromboembolic complications and five patients (4.0%) had lymphocele that required treatment. There was a correlation between the American Society of Anesthesiologists (ASA) physical status classification and postoperative complications (*p*=0.06), but it was not possible to identify statistically significant predictors.

**Conclusion:**

ePLND during radical prostatectomy has a low rate of intraoperative complications and may change prostate cancer staging. Postoperative complications, especially venous thromboembolism and lymphocele, need to be monitored even in the late postoperative period.

## 1. Introduction

Prostate cancer (PC) screening programs have been increasing in the last decades worldwide [1]. Nevertheless, the diagnosis of patients with locally advanced PC, with high risks of extra prostatic extension and lymph node involvement, remains significant. [2]. This group of patients seems to have the greatest benefits from PC treatment, especially due to the possibility of associating other treatment modalities after surgical procedures, such as radiotherapy and hormone blockade therapy, to improve survival [2].

Extended pelvic lymph node dissection (ePLND) has a greater accuracy to detect lymph node dissemination from PC compared to other methods, even with the emergence of new imaging technologies [2–4]. Despite the importance of this procedure for PC staging, its therapeutic role remains unclear [5].

The increase in minimally invasive surgical procedures for PC treatment was accompanied by reduction in the performance of ePLND during radical prostatectomy, probably due to increased morbidity, surgical time, and costs related to the procedure [6]. The measurement of ePLND complication rates is still a challenge and only a few studies used strict criteria for detailing complications [7–11].

The benefits and harms of ePLND for the treatment of PC are controversial and are constantly being studied. This study aims to evaluate risk factors for intra and postoperative complications of ePLND and its morbidity, optimizing the selection of patients who may benefit. Finally, it is worth asking whether we are adequately selecting patients to undergo extended pelvic lymphadenectomy, weighing the oncological benefits against perioperative morbidity.

## 2. Patients and Methods

This is a retrospective cohort evaluating predictors of surgical complications in patients with localized PC and a high risk for lymph node involvement, submitted to radical prostatectomy and ePLND. High risk for the extra-prostatic disease was defined according to D'Amico risk classification (PSA > 20 ng/dl, Gleason score > 7 or digital rectal exam suggesting extra prostatic disease) and/or risk of lymph node involvement greater than 5% according to the Briganti's nomogram, version 2012 [10, 11].

All procedures were performed between 2013 and 2019 using minimally invasive approaches, either laparoscopic or robot-assisted (da Vinci Si system), in two hospitals in Belo Horizonte, MG, Brazil. The operations were performed by a single surgeon with more than 100 cases of experience in radical prostatectomies for each surgical approach, before the beginning of the study.

Bilateral ePLND was performed immediately before radical prostatectomy, in the same surgical procedure. It included the removal of the lymph nodes located along the obturator fossa, internal iliac artery, external iliac artery, and up to the crossing of the ureter with the common iliac artery.

A suction drain was used only in the laparoscopic approach. All patients received venous thromboembolism (VTE) prophylaxis with low molecular weight of heparin during hospitalization. Patients with either a previous history of VTE or two or more risk factors for VTE (BMI > 35 Kg/m^2^, age 75 years or more, family history of VTE) had their prophylaxis extended to 28 days. The postoperative follow-up was performed at7, 30, and 90 days after surgery. All postdischarge outpatient and emergency department visits and hospitals readmissions were recorded.

Clinical and pathological variables such as age, physical status classification of the American Society of Anesthesiologists (ASA), body mass index (BMI), age-adjusted Charlson comorbidity index, total prostate-specific prostate antigen (PSA) value (ng/ml), prostate weight, International Society of Urological Pathology (ISUP) grading system, and type of minimally invasive approach were analyzed. Surgical time, bleeding, and hospitalization time were also evaluated.

Postoperative pathological examinations were performed by two anatomic pathology laboratories, which support the hospitals where the surgeries were performed. The pathological report included the number of lymph nodes removed, the number of lymph nodes with metastasis, extra-prostatic disease identification, surgical margins status, and the pathological TNM Staging System [12]. TNM categories I and II were separated from categories III and IV and subjected to joint studies to improve statistical analysis.

Intraoperative and postoperative complications were evaluated and classified based on Satava[13, 14] and Clavien-Dindo-Strasberg [8, 9, 14] systems, respectively. Postoperative complications were defined as early (≤30 days) or late (>30 days). For the proper notification of complications, Martin's ten criteria were followed [7, 13]. Patients were not routinely investigated for lymphocele. When compressive or infectious symptoms were suspected, computed tomography was performed for diagnosis.

Data were collected by urologists of the institutions involved and recorded in a database made with the software REDCap (Vanderbilt University). This study was approved by the institutional review board of the institutions.

### 2.1. Statistical Analysis

The qualitative variables were presented as absolute and relative frequencies, and the quantitative variables as mean ± standard deviation for those with normal distribution and the median (P25; P75) for those without it. The quantitative variables were submitted to the Shapiro–Wilk normality test. The association between qualitative variables was evaluated by the Chi-Square test, Fisher exact test, and adjusted (Monte Carlo) Chi-Square test for those with more than two categories. The comparison between quantitative variables between two groups was performed via the t-Student or Mann–Whitney tests for independent samples. A multiple logistic regression model was constructed using the backward method. Variables with *p* value < 0.20 were included in the multivariate model. The analyses were performed with the software *R* v.4.0.2 and statistical significance was set at *p* < 0.05.

## 3. Results

Overall, 135 medical records were evaluated and 11 were excluded for the following reasons: performance of concomitant inguinal herniorrhaphy (*n* = 5), postradiotherapy salvage radical prostatectomy (*n* = 3), neoadjuvant PC treatment (*n* = 2), and performance of a super extended pelvic lymph node dissection due to lymph node metastasis suspected in the presacral area. Therefore, 124 cases were analyzed, with a mean age of 66.1 ± 6.8 years and the median BMI of 26.67 (24.3; 28.7) kg/m^2^. ASA 2 score was predominant (71.1%) also age-adjusted Charlson ≤2 (58.2%). The median PSA value was 7.0 (4.5; 12.3) ng/mL. The ISUP rating of 3–5 (69.1%) was the most frequent.

The laparoscopic approach was performed in 60 patients (48.4%), while the laparoscopic robot-assisted was performed in 64 (51.6%). The mean operative time was 149.9 ± 33.3 minutes, and the median length of hospital stay was 50.1 (34.9; 68.0) hours. The median intraoperative bleeding was 275 ml (Table 1). Only one patient was hemotransfused due to the left internal iliac vein injury.

The median number of lymph nodes dissected was 9 [6,12], with 28.2% of lymph nodes positive for metastasis. Extra-prostatic disease was present in 60.5% of the patients, 29.8% of which had an involved seminal vesicle, and 37.1% had a compromised surgical margin. Stage III cancer was the most frequent, with 50 patients (40.3%) (Table 2). Neither the laparoscopic nor robot-assisted approach was determining the factor in the number of lymph nodes removed (*p*=0.391), and it did not interfere in oncological outcomes.

Regarding the intraoperative period, there were 5 complications in the laparoscopic approach (4%) against none in the other arm (*p*=0.024) (Table 3).

Analyzing the postoperative period, 49 complications occurred in 40 patients (32.3%). Nine (7.3%) were considered severe complications (Clavien 3–5). Three (2.4%) patients had VTE, of which two had thrombosis in the lower limb and one had pulmonary thromboembolism. Lymphatic complications directly related to ePLND occurred in 21 patients (16.9%) (Table 4). Five patients (4.0%) had lymphocele that required intervention, and three of them had this complication diagnosed after postoperative day 30 (Table 5). Among severe complications, there were two (1.6%) deaths: one pulmonary aspiration after vomiting due to intestinal obstruction on the postoperative day 4, and one pulmonary thromboembolism on the postoperative day 21 (Table 5).

The most frequent complication was lymphedema of the genital region and lower limbs. In all cases, it resolved spontaneously within 2 weeks without the need for intervention (Table 4).

Postoperative complications were not associated with pre or postoperative ISUP, surgical approach, operative time, blood loss volume, number of lymph nodes removed, presence of lymph node metastasis, extra-prostatic disease, seminal vesicles invasion, compromised surgical margins, or TNM staging (Tables 1 and 2).

No intraoperative variables, such as operative time, blood loss volume, and length of stay, in addition to pathology characteristics were considered risk factors for complications. Intraoperative complications were not a predictor of postoperative complications.

The variables in which *p* value was less than 0.20, namely, the TNM staging and ASA score were evaluated in a multivariate model, and they were not associated with postoperative complications (Table 6).

## 4. Discussion

ePLND, performed concomitantly with radical prostatectomy, is a very distinct step of the procedure, with its specific operative time and complications. The unclear clinical benefits, the increase in operative time, and the risk of complications have made several surgeons to omit it during radical prostatectomy [15, 16]. Yuh et al. in a systematic review demonstrated several case series in which surgeons did not perform ePLND even in high-risk patients [5]. Lestingi et al. published the only randomized clinical trial on this subject and, although the short-term oncological outcome was similar, a subgroup with high-risk patients showed a potential benefit related to biochemical recurrence-free survival [17].

As imaging exams still have a low accuracy for the diagnosis of lymph node metastasis, especially less than 1 centimeter, ePLND remains the gold standard for the lymph node staging in PC [16, 18]. Gandaglia et al. suggested that there was a greater chance to detect lymph node metastasis as the number of lymph nodes removed gets higher [19]. The performance of ePLND brings important information that contributes to prognosis estimation, therefore allowing better selection of those who will benefit from adjuvant treatment. Briganti et al. found an improved survival of patients submitted to adjuvant radiotherapy when there were up to two lymph node metastases [3].

In this study, the mean number of lymph nodes removed was 10.2 ± 4.9. Fossati et al. published a systematic review of the literature, in which 66 studies evaluated 275, 269 patients undergoing different extents of pelvic lymph node dissection. The mean number of resected lymph nodes ranged from 3.5 to 24 lymph nodes [19].

Ploussard et al. suggested that the quality of lymph node dissection cannot be related exclusively to the number of lymph nodes resected. The method of nodal individualization performed in the surgical specimens by the pathologist, in addition to the anatomy of each patient, can interfere with this assessment [18].

Yuh et al. suggested that the extent and quality of lymph node dissection depend on the surgeon skills more than on the type of surgical approach performed [5]. In accordance with the literature, this study showed that the method of minimally invasive procedure, laparoscopic or robot-assisted, did not interfere in the mean number of resected lymph nodes.

In the present study, 28.2% of patients submitted to ePLND had at least one positive lymph node. Therefore, this procedure had a clear impact on decisions involving adjuvant treatment also the follow-up of these patients. This proportion was higher than those reported in the literature, which ranged from 0.13% to 26.2% [5, 18]. The heterogeneity of preoperative staging, including the quality of prostate biopsies, which in this study were largely performed without previous magnetic resonance, may have understated some patients. This hypothesis further reinforces the need for lymph node dissection for the adequate staging of the disease. The latest version of Briganti's nomogram (version 2018) [19], which incorporated magnetic resonance and fusion biopsy findings, seems to minimize understating.

According to the literature, intraoperative complication rates during ePLND are usually low, ranging from 0% to 5% [20]. In this study, all five intraoperative complications occurred in laparoscopic procedures. This fact may be related to the beginning of the learning curve, as the laparoscopic robot-assisted procedures were performed after the pure laparoscopic surgeries. Although the surgeon already had experience in both surgical approaches before the study started, there may have had improvement in the surgical technique of ePLND, as it became more standardized. Eden et al. published a study suggesting that the risk of specific complications related to lymph node dissection decreases after 40 cases [21].

Although some studies do not correlate ePLND with higher postoperative complications [22], most series report increase morbidity as the number of lymph nodes removed increases. The postoperative complication rate of 32.3% in this study was higher than the rate reported in the literature, which ranged from 8.3% to 26.9% [5, 18, 23]. Part of this difference might be explained by the methodological rigor used in the data collection of this study, especially in the evaluation of late complications. Approximately 20% of complications occurred after the postoperative day 30. The use of the Martin's criteria seems to greatly improve the quality of the evaluation of complications [7, 23, 24].

Severe complications rate of 7.3% in the present study was similar to other studies in the literature, and it is often difficult to establish whether the complication was due to ePLND or to radical prostatectomy [18]. Lymphoceles are the most frequent complications specifically related to pelvic lymphadenectomy, especially when it is performed in an extended fashion. The 4% rate of lymphoceles reported in this study was similar to those described in the literature, which ranged from 2.4% to 6.6% [5, 18]. Therefore, missing the ePLND based on its complication does not seem to be appropriate. In addition, as the clinical manifestations of lymphocele often occur after postoperative day 30, the data of this study support patients should be informed about specific warning signs and symptoms of this complication even in the late postoperative period.

The VTE rate demonstrated in this study was higher than those frequently published in the literature, even though the care recommended by most protocols was followed [25]. Recently, Patel et al. published a randomized study that failed to show a reduction in symptomatic VTE with the use of pharmacological prophylaxis. However, the seven patients in this randomized study who presented symptomatic VTE had undergone ePLND [26]. The type of prophylaxis, advantages and disadvantages, and time duration still need to be better established.

Identifying predictors of complications for better selection of candidates for ePLND remains a challenge. As demonstrated in this study, Liss et al. could not show risk factors for complications [27]. Other studies have reported a correlation of the ASA score with complications. Nevertheless, this correlation was probably not significant in this study due to the number of patients analyzed. Agarwal et al. published a study with 3, 317 patients undergoing prostatectomy in which age, Gleason score, and gastroesophageal reflux disease were predictors of complications [28].

Among the limitations of this study, we can enumerate the retrospective design, lack of a control group of patients not submitted to ePLND, and all procedures performed by the same surgeon. Another one was the lack of a standard method for anatomopathological assessment of the lymph nodes. Besides, the limited number of patients may have impaired the identification of predictors for complications.

## 5. Conclusions

ePLND during radical prostatectomy has a low rate of intraoperative complications. Postoperative complications, especially VTE and lymphocele, need to be monitored even in the late postoperative period. The authors did not identify any predictive factor of ePLND's complications, supporting the concept that, when properly indicated, lymph node dissection should be performed regardless of the minimal invasive surgical approach.

## Figures and Tables

**Table 1:** Univariate analysis of clinical-pathological variables in the pre and intraoperative period and the occurrence of postoperative complications.

Variables	Presence of postoperative complications		*p * ** -**value	Total *(n* *=* *124)*
	Absent *(n* *=* *84)*	Present *(n* *=* *40)*		
***Preoperative clinical-pathological variables***				
Age (years)	66.2 ± 6.9	65.8 ± 6.7	0.737^T^	66.1 ± 6.8
BMI^*∗*^ (Kg/m2) (*n* = 108)	26.5 (24.1; 28.7)	27.1 (24.8; 29.3)	0.423^W^	26.6 (24.3; 28.7)
ASA^*∗*^ (*n* = 121)			0.065^Q^	
1	20 (24.4%)	5 (12.8%)		25 (20.7%)
2	53 (64.6%)	33 (84.6%)		86 (71.1%)
3	9 (11%)	1 (2.6%)		10 (8.3%)
Charlson^*∗*^ (*n* = 122)		0.450^M^		
≤2	47 (57.3%)	24 (60.0%)		71 (58.2%)
3–4	31 (37.8%)	16 (40.0%)		47 (38.5%)
≥5	4 (4.9%)	0 (0.0%)		4 (3.3%)
PSA^*∗*^ (ng/mL) (*n* = 122)	7.2 (4.7; 12.2)	6.2 (4.3; 14.1)	0.961^W^	7 (4.5; 12.3)
Prostate weight	41.0 (31.5; 57.0)	44 (33.0; 54.5)	0.775^W^	41.5(32.0; 55.0)
Preoperative ISUP^*∗*^ (*n* = 123)		0.326^Q^		
1–2	28 (33.7%)	10 (25.0%)		38 (30.9%)
3–5	55 (66.3%)	30 (75.0%)		85 (69.1%)
Surgical approach			0.743^Q^	
Laparoscopic	42 (50%)	18 (45%)		60 (48.4%)
Robot-assisted	42 (50%)	22 (55%)		64 (51.6%)

***Intraoperative variables***				
Operative time (minutes)^*∗*^ (*n* = 121)	149.3 ± 31.5	151 ± 36.8	0.821^T^	149.9 ± 33.1
Estimated bleeding (mL)^*∗*^ (*n* = 120)	300 (150; 400)	250 (150; 380)	0.993^W^	275 (150; 390)
Intraoperative complications	4 (4.8%)	1 (2.5%)	1.000^F^	5 (4%)
Length of hospital stay (hours)	50.1 (37.8; 55.2)	50.5 (37.0; 98.8)	0.318^W^	50.1 (34.9; 68.0)

^T^ t-Student test for independent samples,^W^ Mann–Whitney test,^Q^ Chi-Square test,^M^ adjusted (Monte Carlo) Chi-square,^F^ Fisher exact test. Continuous data expressed in mean ± standard deviation for data with normal distribution and median (P25; P75) for data without normal distribution. ^*∗*^*n* is less than the total number of patients due to missing data.

**Table 2:** Univariate analysis of anatomopathological characteristics and the occurrence of postoperative complications.

Variables	Presence of postoperative complications		*p*-value	Total (*n* = 124)
	Absent *(n = 84)*	Present *(n = 40)*		
*Anatomopathological variables*				
Postoperative ISUP		0.949^Q^		
Differentiated	32 (38.1%)	15 (37,5%)		47 (37,9%)
Undifferentiated	52 (61,9%)	25 (62,5%)		77 (62,1%)
Number of lymph nodes removed^*∗*^ (*n* = 121)	9 (6; 12)	9 (7; 13)	0.934^W^	9 (6; 12)
Positive lymph nodes	26 (31%)	9 (22,5%)	0.445^Q^	35 (28,2%)
Extra-prostatic disease	49 (58.3%)	26 (65%)	0.608^Q^	75 (60,5%)
Positive seminal vesicles	23 (27.4%)	14 (35%)	0.511^Q^	37 (29,8%)
Positive margins	34 (40.5%)	12 (30%)	0.352^Q^	46 (37,1%)
TNM Staging			0.078^M^	
I	5 (6.0%)	0 (0,0%)		5 (4,0%)
II	25 (29.8%)	9 (22,5%)		34 (27,4%)
III	28 (33.3%)	22 (55,0%)		50 (40,3%)
IV	26 (31.0%)	9 (22,5%)		35 (28,2%)
Grouped staging			0.138^Q^	
Initial (I + II)	30 (35.7%)	9 (22,5%)		39 (31,5%)
Advanced (III + IV)	54 (64.3%)	31 (77,5%)		85 (68,5%)

^W^ Wilcoxon Mann–Whitney test, ^Q^ Chi-Square test, ^M^ adjusted (Monte Carlo) Chi-square test. ^*∗*^*n* is less than the total number of patients because the number of lymph nodes removed in three patients was not described.

**Table 3:** Intraoperative complications, according to surgical approach, treatment, and Satava severity complication classification (*n* = 5).

Intraoperative complications	Surgical approach	Treatment	Satava Classification (grade)
Left ureteral injury	Laparoscopic	Uretero-ureteral anastomosis	2
Rectus lesion	Laparoscopic	Suture	2
Left internal iliac vein injury	Laparoscopic	Suture	2
Lesion of the bladder dome on the left	Laparoscopic	Suture	2
Needle break (needle fragment permanence in the pelvis)	Laparoscopic	Conservative	1

**Table 4:** Description of complications according to type, severity, and moment of occurrence in a cohort of 124 patients undergoing extended pelvic lymph node dissection.

Complications	Number of occurrence^*∗*^ (*n* = 40)	Incidence (%)
Venous thromboembolism	3	(2.4%)
Clinical lymphocele	5	(4.0%)
Lymphedema	17	(13.7%)
Genitourinary	11	(8.9%)
Gastrointestinal	5	(4.0%)
Bleeding	2	(1.6%)
Abdominal wall	2	(1.6%)
Neuromuscular	2	(1.6%)
Cardiovascular	2	(1.6%)
**Total**	49	—
Clavien		
1–2	40	(32.3%)
3–5	09	(7.3%)
Moment of occurrence		
During hospitalization	14	(28.6%)
<30 days	25	(51.0%)
>30 days	10	(20.4%)

^*∗*^Patients who had more than one complication were counted more than once.

**Table 5:** Severe postoperative complications (Clavien 3–5), a moment of occurrence and treatment performed (*n* = 9).

Complication	Moment of occurrence	Treatment
Urinary sepsis	<30 days	Clinical support
Infected lymphocele	3 months	Percutaneous drainage
Incisional hernia	2 months	Surgical treatment
Infected lymphocele	2 months	Percutaneous drainage
Infected lymphocele	<30 days	Percutaneous drainage
Infected lymphocele	8 months	Laparoscopic marsupialization
Venous thromboembolism	<30 days	Death
Pulmonary aspiration during decompressive colonoscopy	During hospitalization	Death
Lymphocele and urinary fistula	<30 days	Laparoscopic drainage

**Table 6:** Multiple logistic regression model with factors associated with postoperative complications in patients undergoing extended pelvic lymph node dissection.

Variables	OR	95% confidence interval for OR		*p*-value
		Minimum	Maximum	
ASA				
ASA 1	1.00	—	—	
ASA 2	2.32	.786	6.85	0.128
ASA 3	0.47	.047	4.65	0.517
**Staging**				
I-II	1.00	—	—	
III-IV	1.84	0.732	4.63	0.194

ASA = American Society of Anesthesiologists; OR = odds ratio.

## Data Availability

Data supporting the results of this study are available upon request to the author for correspondence.

## References

[1] Loeb S., Bjurlin M. A., Nicholson J. (2014). Overdiagnosis and overtreatment of prostate cancer. *European Urology*.

[2] Briganti A., Karnes R. J., Gandaglia G. (2015). Natural history of surgically treated high-risk prostate cancer. *Urologic Oncology: Seminars and Original Investigations*.

[3] Briganti A., Abdollah F., Nini A. (2012). Performance characteristics of computed tomography in detecting lymph node metastases in contemporary patients with prostate cancer treated with extended pelvic lymph node dissection. *European Urology*.

[4] Bianchi L., Gandaglia G., Fossati N. (2017). Pelvic lymph node dissection in prostate cancer: indications, extent and tailored approaches. *Urologia Journal*.

[5] Yuh B., Artibani W., Heidenreich A. (2014). The role of robot-assisted radical prostatectomy and pelvic lymph node dissection in the management of high-risk prostate cancer: a systematic review. *European Urology*.

[6] Nocera L., Sood A., Dalela D. (2018). Rate and extent of pelvic lymph node dissection in the US prostate cancer patients treated with radical prostatectomy. *Clinical Genitourinary Cancer*.

[7] Martin R. C. G., Brennan M. F., Jaques D. P. (2002). Quality of complication reporting in the surgical literature. *Annals of Surgery*.

[8] Clavien P. A., Sanabria J. R., Strasberg S. M. (1992). Proposed classification of complications of surgery with examples of utility in cholecystectomy. *Surgery*.

[9] Dindo D., Demartines N., Clavien P. A. (2004). Classification of surgical complications: a new proposal with evaluation in a cohort of 6336 patients and results of a survey. *Annals of Surgery*.

[10] Hansen J., Rink M., Bianchi M. (2013). External validation of the updated briganti nomogram to predict lymph node invasion in prostate cancer patients undergoing extended lymph node dissection. *The Prostate*.

[11] Briganti A., Blute M. L., Eastham J. H. (2009). Pelvic lymph node dissection in prostate cancer. *European Urology*.

[12] Sobin Mkg L. H., Wittekind C. (2009). *Classification of Malignant Tumours*.

[13] Satava R. M. (2005). Identification and reduction of surgical error using simulation. *Minimally Invasive Therapy & Allied Technologies*.

[14] Strasberg S. M., Linehan D. C., Hawkins W. G. (2009). The accordion severity grading system of surgical complications. *Annals of Surgery*.

[15] Zorn K. C., Katz M. H., Bernstein A. (2009). Pelvic lymphadenectomy during robot-assisted radical prostatectomy: assessing nodal yield, perioperative outcomes, and complications. *Urology*.

[16] Touijer K. A., Ahallal Y., Guillonneau B. (2013). Indications for and anatomical extent of pelvic lymph node dissection for prostate cancer: practice patterns of uro-oncologists in North America. *Urologic Oncology: Seminars and Original Investigations*.

[17] Lestingi J., Guglielmetti G. B., Trinh Q.-D. (2020). Extended vs. limited pelvic lymph node dissection during radical prostatectomy for intermediate- and high-risk prostate cancer: early oncological outcomes from a randomized phase 3 trial. *SSRN Electronic Journal*.

[18] Ploussard G., Briganti A., De La Taille A. (2014). Pelvic lymph node dissection during robot-assisted radical prostatectomy: efficacy, limitations, and complications - a systematic review of the literature. *European Urology*.

[19] Gandaglia G., Ploussard G., Valerio M. (2019). A novel nomogram to identify candidates for extended pelvic lymph node dissection among patients with clinically localized prostate cancer diagnosed with magnetic resonance imaging-targeted and systematic biopsies. *European Urology*.

[20] Musch M., Klevecka V., Roggenbuck U., Kroepfl D. (2008). Complications of pelvic lymphadenectomy in 1, 380 patients undergoing radical retropubic prostatectomy between 1993 and 2006. *The Journal of Urology*.

[21] Eden C. G., Zacharakis E., Bott S. (2013). The learning curve for laparoscopic extended pelvic lymphadenectomy for intermediate- and high-risk prostate cancer: implications for compliance with existing guidelines. *BJU International*.

[22] Yuh B. E., Ruel N. H., Mejia R., Novara G., Wilson T. G. (2013). Standardized comparison of robot-assisted limited and extended pelvic lymphadenectomy for prostate cancer. *BJU International*.

[23] Cheng W. M., Lin T. P., Lin C. C. (2014). Standardized report for early complications of radical prostatectomy. *Journal of the Chinese Medical Association*.

[24] Löppenberg B., Noldus J., Holz A., Palisaar R. J. (2010). Reporting complications after open radical retropubic prostatectomy using the martin criteria. *The Journal of Urology*.

[25] Chair K. A. O. T., Cartwright R., Gould M. K. (2020). *UAE Guidelines*.

[26] Patel H. D., Faisal F. A., Trock Bj (2020). Effect of pharmacologic prophylaxis on venous thromboembolism after radical prostatectomy: the preventer randomized clinical trial. *European Urology*.

[27] Liss M. A., Palazzi K., Stroup S. P., Jabaji R., Raheem O. A., Kane C. J. (2013). Outcomes and complications of pelvic lymph node dissection during robotic-assisted radical prostatectomy. *World Journal of Urology*.

[28] Agarwal P. K., Sammon J., Bhandari A. (2011). Safety profile of robot-assisted radical prostatectomy: a standardized report of complications in 3317 patients. *European Urology*.

